# Long-Term Outcomes After Hepatectomy for Alveolar Echinococcosis in Immunosuppressed Patients

**DOI:** 10.3390/pathogens15070756

**Published:** 2026-07-19

**Authors:** Djana Rrupa, Christelle Kaiser, Emmanuel Melloul, Emilie Uldry, Nermin Halkic, Guido Beldi, Severin Gloor, Anja Lachenmayer, Gaëtan-Romain Joliat

**Affiliations:** 1 Department of Visceral Surgery, Lausanne University Hospital CHUV, University of Lausanne (UNIL), 1011 Lausanne, Switzerland; djana.rrupa@gmail.com (D.R.);; 2 Department of Visceral Surgery and Medicine, Inselspital—Bern University Hospital, University of Bern, 3010 Bern, Switzerland; 3 Department of General, Visceral, and Transplant Surgery, Muenster University Hospital, 48149 Muenster, Germany

**Keywords:** alveolar echinococcosis, *Echinococcus multilocularis*, liver resection, immunosuppressed patients, outcomes

## Abstract

Long-term outcomes after hepatectomy for alveolar echinococcosis (AE) in immunosuppressed patients remain poorly investigated. This study evaluated recurrence rates and recurrence-free/overall survivals (RFS/OS) after AE resection in immunocompromised and immunocompetent patients. Consecutive patients operated for liver AE in two university hospitals were retrospectively collected (2000–2021). Immunosuppressed patients were defined as patients who had a reduced ability to fight infection due to certain diseases or treatments. 195 patients had hepatectomy for liver AE. Preoperative albendazole was given in 111 cases (57%). Fifty-two patients (27%) were considered immunosuppressed. Patients in the immunosuppressed and immunocompetent cohorts had similar preoperative characteristics. Recurrences occurred in 10 patients (immunosuppressed group: 4, non-immunosuppressed group: 6) within a median follow-up of 58 months (95%CI 48–68). No significant differences in RFS and OS were found between the immunosuppressed and immunocompetent groups (212 vs. 206 months, *p* = 0.625 and 236 vs. 210 months, *p* = 0.282). Two-year recurrence rates were 0% in the immunosuppressed cohort and 1% (1/142) in the immunocompetent patients (*p* = 0.466). Absence of preoperative albendazole and lesion size were predictive of recurrence after hepatectomy. Immunosuppression was not found to be a risk factor for recurrence (HR 1.4, *p* = 0.626). In this bicentric study, immunocompromised patients did not have significantly different recurrence rates, RFS, and OS than immunocompetent patients.

## 1. Introduction

Alveolar echinococcosis (AE) is a rare but severe zoonosis that mimics a slow-growing malignant tumor [1,2,3]. The definitive and intermediate hosts of *Echinococcosis multilocularis* are foxes (less commonly, dogs and cats) and rodents, respectively. Foxes produce thousands of eggs that are released in their feces. The primary infection pathway of AE is by oral ingestion of *E. multilocularis* eggs. These eggs most frequently reach the liver via the portal or lymphatic system. They then transform into the larval stage (metacestodes). The final location of the larvae is the liver in most cases, while only a small minority migrate to other body sites (especially the lungs, brain, bones, muscles, or kidneys) [4,5].

AE is the most prevalent food-borne parasitic disease in Europe and is classified second worldwide in terms of mortality [6,7]. AE is endemic in Europe, Central Asia, Northern Japan, and Russia. In Europe, the endemic areas are in central Europe (e.g., Southern Germany, Switzerland, Western Austria, Eastern France, or Croatia). In these areas, a rising incidence of human infections has been reported, which is most likely associated with the increase in the infected urban and rural fox populations [8,9] and with improved diagnosis due to modern imaging techniques [10]. This may also be attributable to an increased number of immunosuppressed human hosts [11].

The current literature suggests that immunosuppression may favor parasite growth and disease progression, which can represent a challenge for treatment and follow-up [12].

Scientific evidence remains scarce about this concern, while the incidence of AE in endemic regions and the use of immunosuppressive therapies is growing considerably.

Given these considerations, patient immunosuppression may not only contribute to the rising incidence of AE but also influence postoperative outcomes, particularly in terms of recurrence-free survival (RFS) and overall survival (OS). The present study aimed to evaluate these outcomes by comparing immunosuppressed and immunocompetent patients undergoing surgery for hepatic AE.

## 2. Methods

### 2.1. Study Design and Participants

This cross-sectional, bicentric retrospective study included all consecutive patients who underwent surgery for hepatic AE between January 2000 and December 2021 at the Lausanne University Hospital (CHUV) and at the University Hospital of Bern (Inselspital).

Inclusion criteria were patients > 18 years old who underwent surgery for hepatic AE at CHUV or Inselspital during the study period.

Patients were excluded if they refused the use of their data for research purposes or if they underwent palliative/R2 resections (incomplete resection with macroscopic residual disease).

### 2.2. Objectives and Outcomes

The main objective of the present study was to evaluate the postoperative outcomes of immunosuppressed patients undergoing curative surgery for hepatic AE, in comparison with immunocompetent patients.

The primary endpoint was the recurrence rates of AE in immunosuppressed versus immunocompetent patients who underwent hepatectomy, assessed at 1, 2, and 5 years after surgery.

The secondary endpoints included RFS, OS, and postoperative 90-day morbidity in immunosuppressed versus immunocompetent patients who underwent hepatectomy for AE. Moreover, predictive factors for recurrence were assessed.

### 2.3. Definitions

Immunosuppression is routinely defined as a reduced ability to combat infections and other diseases, resulting from specific conditions or treatments [13]. In the present study, patients were considered immunosuppressed if they had human immunodeficiency virus (HIV), cancer, uncontrolled diabetes, severe malnutrition, or were undergoing immunosuppressive treatments, including anticancer drugs, corticosteroids, stem cell therapy, or immunosuppressants following organ transplantation, at the time of diagnosis, surgery, or during postoperative follow-up.

Complications were defined by the Clavien–Dindo classification [14]. Moreover, the Comprehensive complication index was calculated to account for the entire burden of morbidity per patient [15].

### 2.4. Statistical Analysis

Data were summarized with median and interquartile range (IQR) and with number and percentage based on the variable types. Comparisons between both groups were made using the Mann–Whitney U-test for continuous data and the chi-square test for categorical data. Supplementary analyses for group comparisons were performed using propensity score matching with a caliper of 0.1. Matching criteria were age, preoperative albendazole, lesion size, presence of extrahepatic metastasis, and number of resected segments.

The primary endpoints (recurrence rates) were compared in both groups at 1, 2, and 5 years postoperatively using chi-square tests.

OS and RFS were calculated using Kaplan–Meier curves. Survival in both groups was compared using the log-rank test. Predictors of recurrence were calculated using a multivariable Cox regression analysis. Only variables with *p* < 0.1 on univariable analysis were included in the multivariable analysis.

The median follow-up time was calculated using the inverse Kaplan–Meier method. All statistics were two-sided, and *p*-values inferior to 5% were considered statistically significant. All statistical analyses were performed using SPSS 29.0 for Mac OS X (IBM, Chicago, IL, USA).

### 2.5. Ethics

The study was approved by both the Cantonal Ethics Committee of Vaud and the Cantonal Ethics Committee of Bern. All included patients provided their general consent to research (i.e., consent for data usage and informed consent). The authors declare that they have no conflicts of interest.

## 3. Results

A total of 195 patients were included in the study: 98 from Bern and 97 from Lausanne. The median age and body mass index of the entire cohort were 58 (IQR 45–68) and 24 kg/m^2^ (IQR 21.2–26.6), respectively. A hundred and two women (52%) and 93 men (48%) were included. Fifty-two patients (27%) were considered immunosuppressed.

### 3.1. Comparisons Between Immunosuppressed vs. Immunocompetent Patients

No difference between the two groups was found, except for the number of patients with controlled and uncontrolled diabetes mellitus, which was higher in the immunosuppressed group, as expected according to our definition of immunosuppression (19% vs. 1%, *p* < 0.001). Preoperative characteristics of immunosuppressed and immunocompetent patients are depicted in Table 1.

In patients without immunosuppression, major hepatectomies were performed more frequently. The median number of resected segments was therefore higher in this group (Table 2).

Regarding the postoperative outcomes, higher rates of hemorrhage and surgical-site infection were found in the immunosuppressed group. Postoperative results are summarized in Table 3.

**Figure 1:**
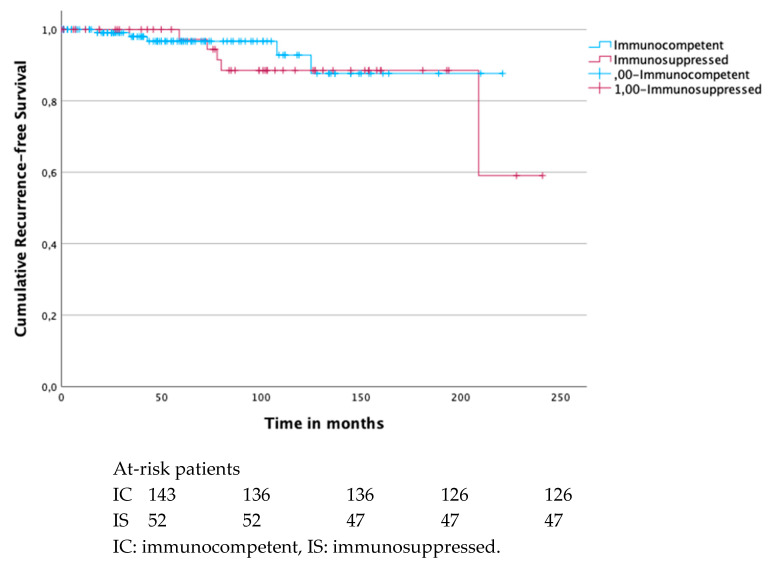
The Kaplan–Meier curves of recurrence-free survival in the immunocompetent and immunosuppressed groups (mean survival: 206 months, 95% CI 191–219 vs. 212 months, 95% CI 190–235, *p* = 0.625).

**Figure 2:**
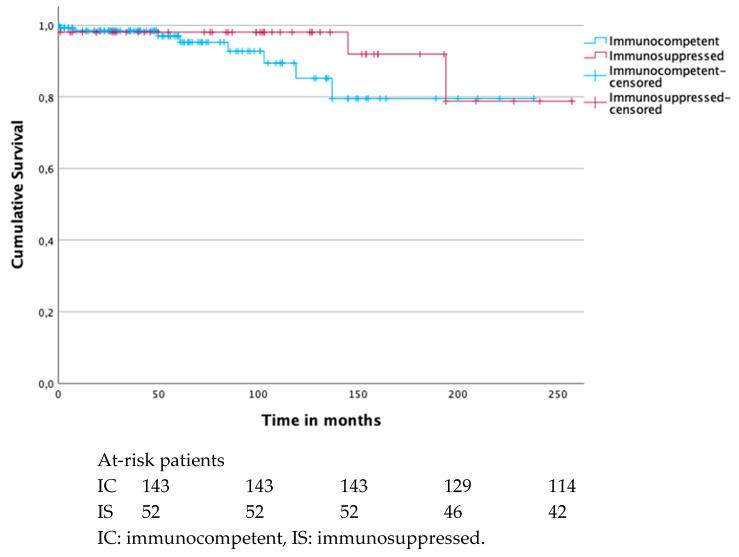
The Kaplan–Meier curves of overall survival in the immunocompetent and immunosuppressed groups (mean survival: 210 months, 95% CI 190–229 vs. 236 months, 95% CI 216–258, *p* = 0.282).

Recurrence occurred in 4 patients in the immunosuppressed group (2 patients had intrahepatic recurrences, and 2 patients had both intra- and extrahepatic recurrences) and in 6 patients in the non-immunosuppressed group (5 patients with intrahepatic recurrences and 1 patient with extrahepatic recurrence) within a median follow-up of 58 months (95% CI 48–68). No differences in RFS and OS were found between the immunosuppressed and immunocompetent groups (Figure 1 and Figure 2). During follow-up, 6 patients died in the immunocompromised group due to leukemia (1), stroke (1), cancer (1), or unknown causes (3), while in the immunocompetent group, 4 patients died due to cancers (2) or unknown causes (2). All deaths were non-AE-related.

One-year, two-year, and five-year recurrence rates were 0%, 0%, and 2% (1/52 at 59 months) in the immunosuppressed cohort and 0% (*p* = 1), 1% (1/142, *p* = 0.466), and 2% (3/142, *p* = 0.934) in the immunocompetent patients, respectively.

On multivariable analysis, absence of preoperative albendazole and lesion size were predictive of recurrence after hepatectomy (Table 4). Immunosuppression was not found as a risk factor for recurrence (HR 1.4, *p* = 0.626).

### 3.2. Immunosuppression Group (n = 52)

The most frequent types of immunosuppression were cancers (n = 15), autoimmune diseases (n = 15), specific immunosuppressive treatments (e.g., for transplantation, n = 11), HIV infection (n = 4), uncontrolled diabetes (n = 3), recurrent infectious problems (n = 3), and inflammatory bowel disease (n = 1). The median postoperative albendazole duration was 21 months (IQR 4–24) in the immunosuppression group and 24 months (IQR 12–24) in the immunocompetent group.

### 3.3. Recurrences

Regarding the 10 patients who had a recurrence, 7 were intrahepatic only, and 3 (2 patients in the immunosuppressed group and 1 in the immunocompetent group) were both intra- and extrahepatic, with involvement of the adrenal gland (1 case), stomach (1 case), and diaphragm and peritoneum (1 case). The median time to recurrence was 69 months, and the median size of the recurrent lesion was 30 mm. Postoperative outcomes of patients who had a recurrence are depicted in Supplementary Table S1.

Among the 4 immunosuppressed patients who had a recurrence, 1 had Sjögren syndrome, 2 had lupus, and 1 had HIV infection. None of them had preoperative albendazole treatment. Two patients were classified as PNM stage I, one as stage III, and one as stage IV (omental and peritoneal lesions).

### 3.4. Sensitivity Analysis

If cancer patients and patients with uncontrolled diabetes are removed from the definition of immunosuppression, the number of immunosuppressed patients is 34. Using that definition of immunosuppression, mean RFS and OS were also similar in both groups (RFS: immunocompetent group: 207, 95% CI 190–225 vs. immunosuppression group: 189 months, 95% CI 151–227, *p* = 0.256 and OS: 222, 95% CI 206–237 vs. 211 months, 95% CI 175–246 *p* = 0.084).

### 3.5. Propensity Score Matching

After propensity score matching (46 patients in each group), no differences were found in preoperative and intraoperative characteristics of patients and postoperative outcomes (Supplementary Table S2). No differences were found in mean RFS (217, 95% CI 196–239 vs. 206, 95% CI 191–219, *p* = 0.207) and mean OS (218, 95% CI 185–242 vs. 211 months, 95% CI 173–250, *p* = 0.255).

### 3.6. Subgroup Analysis for Major Hepatectomy

Among patients who underwent major hepatectomy (n = 111), recurrence rates at 1, 2, and 5 years were 0% vs. 0% (*p* = 1), 0% vs. 0% (*p* = 1), and 1/23 (4%) vs. 2/88 (2%, *p* = 0.549) in the immunosuppressed and non-immunosuppressed groups, respectively. Mean RFS and OS were similar in both groups (RFS: immunocompetent group: 179, 95% CI 161–198 vs. immunosuppression group: 141 months, 95% CI 110–173, *p* = 0.063 and OS: 212, 95% CI 189–235 vs. 208 months, 95% CI 167–250, *p* = 0.244).

## 4. Discussion

The association between immunosuppression and outcomes of patients operated for AE has been poorly described in the literature. The incidence of AEs and immunosuppressive therapies is growing considerably, and a better understanding of the immunosuppression impact is needed [11]. The present study analyzed the long-term outcomes of immunosuppressed patients undergoing surgical treatment and compared them to an immunocompetent group over 20 years. No significant differences between the two groups were found in terms of RFS, OS, and recurrence rates. The only predictive factors of RFS were preoperative albendazole treatment and lesion size. Immunosuppression was not found to be a risk factor for recurrence (HR 1.4, *p* = 0.626).

The current literature supports the results of the present study [16]. Deibel et al., in a retrospective cohort study, analyzed the clinical characteristics and outcomes of AE in patients with immunosuppression-associated conditions at a Swiss referral center (n = 189) from 2000–2021. An increasing overall incidence of AE was shown over time in immunosuppressed and non-immunosuppressed patients. Nonetheless, immunosuppressed patients were typically older, more likely to have AE diagnosed incidentally, with smaller hepatic lesions, and had lower seropositivity for anti-Em18 [16]. Despite these differences, clinical outcomes were largely favorable in the immunosuppressed group, suggesting that AE does not necessarily behave more aggressively in immunocompromised hosts. These findings corroborate the results of our study, showing no difference in survival after hepatectomy in both groups. On the other hand, Lachenmayer et al. [17] showed a significant increase in AE incidence among immunosuppressed patients. They showed that, despite the increasing incidence, outcomes are significantly improved by treatment with radical R0 surgery and long-term antiparasitic treatment. Of note, this previous study suggested that immunosuppression was associated with AE incidence and crude overall prognosis in an entire AE population (not only operated patients).

However, the impact of immunosuppression on AE evolution remains complex and is probably dependent on the degree and duration of immunosuppression. Autier et al. [18] emphasized that immunocompromised patients may present with altered host-parasite interaction pathways, leading to more extensive or atypical forms of AE, with potential diagnostic and therapeutic challenges, including delayed diagnosis, difficulties achieving complete surgical control, and the need for prolonged antiparasitic therapy. Nevertheless, these observations mainly concern the overall AE population, including patients managed conservatively or with advanced disease, and do not specifically address outcomes after curative-intent hepatectomy. In this context, our findings suggest that when patients are appropriately selected for surgery and receive adequate perioperative albendazole therapy, immunosuppression alone may not represent an independent risk factor for recurrence or impaired long-term survival.

The multivariable analysis of the present study showed that preoperative albendazole treatment and lesion size were predictors of the recurrence rate, but cautious interpretation should be made because few recurrences occurred during the study period. Moreover, the previous study by Lachenmeyer et al. [17] and the present study should be seen as complementary, as the latter focused only on assessing whether immunosuppression was associated with recurrence and survival in an AE cohort of patients who underwent non-palliative hepatectomy and not patients treated conservatively, unresectable or palliative patients.

Interestingly, the present analysis showed that the immunocompromised group was associated with a higher risk of postoperative hemorrhage and surgical site infections, even though more major hepatectomies were performed in the immunocompetent group with no significant differences in length of stay, reoperation, or readmission. These results are consistent with the literature findings, showing immunosuppression as a risk factor for surgical site infections and for higher hemorrhage risk due to direct bone marrow suppression, immune-mediated mechanisms, or increased susceptibility to infections [13,19].

AE remains a challenging diagnosis, especially in immunocompromised patients. The large analysis of the French registry made by Cauchet et al. [11] showed that AE in the immunosuppressed group had a significantly higher proportion of incidental diagnoses (78% vs. 42%, *p* < 0.001) and presented more frequently with early-stage disease. These data can explain the similar favorable long-term clinical outcomes as in the immunocompetent group, suggesting that if AE is treated early in these patients, the role of immunosuppression might be less deleterious to disease evolution. While the stages of disease were similar in our study, more minor hepatectomies were performed in the immunosuppressed group, potentially reflecting more favorable liver locations.

It was unexpected and counterintuitive to find that OS were not significantly different between immunocompetent and immunocompromised patients. OS in patients with AE is often driven by other competing causes of death, especially in immunosuppressed patients. The risk of a type II error, as the cohort of immunocompromised patients was of moderate size, potential selection bias, residual confounding factors, or the broad definition of immunosuppression could be reasons explaining this finding. It should be noted that most patients did not die from AE but from another cause in the present cohort.

The duration of postoperative albendazole was usually based on the resection margin status. In the case of R0 resection, patients received albendazole for 24 months after surgery, while in the case of R1 resection, lifelong or long-term treatment was given. Intolerance to benzimidazole was a reason to interrupt the postoperative treatment. Data about albendazole intolerance were not available.

This study has some limitations that need to be mentioned. The retrospective cross-sectional design has inherent limitations and risks of bias (missing data or errors during data collection). Moreover, the definition of immunosuppression varies in the literature. The retained definition might not be selective enough, but the authors wanted to have a broad inclusion of patients. Finally, it was not possible to retrospectively collect data on the duration of immunosuppression and whether the immunosuppression was present pre- or postoperatively, or both, which could have influenced the results. The rate of recurrences was low (10/195), which precluded the inclusion of a large number of variables in the multivariable analysis. However, this study provides detailed clinical data and outcomes from a large cohort of immunosuppressed AE patients who were treated with surgery and albendazole.

In conclusion, the present study, focusing on a selected cohort of surgically treated AE patients, emphasized that patients who were immunocompromised at the diagnosis or after diagnosis did not have different long-term outcomes after resection compared with immunocompetent patients, with no differences in RFS, OS, or recurrence rates. Furthermore, immunosuppression was not found to be a risk factor for recurrence. Further investigations, especially as the numbers of recurrences and events in each group were relatively low in the present cohort, are needed to study the specific host immune response to *E. multilocularis* in immunocompromised and immunocompetent patients to better understand the underlying molecular and immunological mechanisms.

## Figures and Tables

**Table 1:** Preoperative characteristics of the patients included in the study based on their immunosuppression status.

	Immunosuppressed n = 52	Non Immunosuppressed n = 143	*p*-Value
Age, years	62 (51–71)	57 (41–67)	**0.022**
Women/men	27 (52%)/25 (48%)	75 (52%)/68 (48%)	0.948
Body mass index, kg/m^2^	25 (22–28)	24 (21–26)	0.217
ASA scores I–II III–IV	35 (67%) 17 (33%)	109 (76%) 34 (24%)	0.210
Cirrhosis	1 (2%)	1 (1%)	0.453
Diabetes mellitus	10 (19%)	1 (1%)	**<0.001**
Positive serology	45 (87%)	125 (87%)	0.872
Preoperative albendazole *	28 (54%)	83 (58%)	0.601
Largest AE lesion on CT, cm	6 (3–9)	6 (4–9)	0.293
Synchronous extrahepatic lesion **	8 (15%)	20 (14%)	0.805

Values are expressed as the median with the interquartile range or the number with the percentage. Significant *p*-values appear in bold. ASA: American Society of Anesthesiologists, AE: alveolar echinococcosis, and CT: computed tomography. * Preoperative albendazole was started at least four weeks before surgery. ** For the 8 immunosuppressed patients: 3 lungs, 2 peritoneal, 1 spleen, and 2 without specification. For the 20 immunocompetent patients: 4 lungs, 2 brains, 3 peritoneal, 2 adrenals, 2 spleens, and 7 without specification. Intra-abdominal lesions were resected at the same time as hepatectomy.

**Table 2:** Intraoperative details of the patients included in the study based on their immunosuppression status.

	Immunosuppressed n = 52	Non Immunosuppressed n = 143	*p*-Value
Bilobar disease	18 (35%)	48 (34%)	0.891
Minor hepatectomy	29 (56%)	55 (38%)	**0.031**
Major hepatectomy	23 (44%)	88 (62%)	**0.031**
Laparotomy	42 (81%)	117 (82%)	0.867
Laparoscopy	10 (19%)	26 (18%)	0.867
Caudate lobe resection	4 (8%)	19 (13%)	0.284
Number of resected segments	2 (2–4)	4 (2–4)	**0.020**
Operation time, min	198 (165–280)	254 (171–334)	0.056
Biliodigestive anastomosis	6 (12%)	26 (18%)	0.268
Intraoperative blood loss, mL	450 (400–800)	600 (300–1000)	0.621
Need of RBC transfusion	7 (13%)	11 (8%)	0.218

Values are expressed as the median with the interquartile range or the number with the percentage. Significant *p*-values appear in bold. RBC: red blood cell.

**Table 3:** Postoperative outcomes for the patients included in the study based on their immunosuppression status.

	Immunosuppressed n = 52	Non Immunosuppressed n = 143	*p*-Value
Largest lesion on pathology, cm	6 (3–9)	7 (4–9)	0.127
Number of lesions	1 (1–2)	1 (1–2)	0.739
Postoperative albendazole	48 (92%)	125 (87%)	0.339
PNM stages I II III IV	26 (50%) 7 (13%) 11 (20%) 8 (17%)	61 (43%) 15 (10%) 46 (32%) 21 (15%)	0.641
R0/R1 resections	40 (77%)/12 (23%)	100 (70%)/43 (30%)	0.337
Complications Minor Major	24 (46%) 14 (27%) 10 (19%)	68 (48%) 28 (20%) 40 (28%)	0.863 0.270 0.216
90-day mortality	1 (2%)	0	0.453
CCI	0 (0–26.9)	0 (0–26.9)	0.979
Biliary leak	4 (8%)	25 (17%)	0.089
Hemorrhage	5 (10%)	2 (1%)	**0.006**
SSI	6 (12%)	5 (3%)	**0.031**
Liver failure	2 (4%)	3 (2%)	0.495
Length of stay, days	10 (6–14)	10 (7–14)	0.737
Reoperation	4 (8%)	10 (7%)	0.867
Readmission	5 (10%)	17 (12%)	0.657

Values are expressed as the median with the interquartile range or the number with the percentage. Significant *p*-values appear in bold. CCI: comprehensive complication index, SSI: surgical site infection, R0: complete resection with microscopically negative margins, and R1: complete macroscopic resection with microscopically positive margins.

**Table 4:** Multivariable Cox regression of preoperative predictors of alveolar echinococcosis recurrence after surgery.

	Univariable, HR (95% CI)	*p*-Value	Multivariable, HR (95% CI)	*p*-Value
Age (years) *	1.0 (1.0–1.0)	0.807		
Immunosuppression	1.4 (0.4–4.8)	0.626		
Preoperative albendazole	0.2 (0.1–1.1)	0.065	0.1 (0.1–1.0)	**0.045**
Lesion size on CT (cm) *	1.0 (1.0–1.0)	0.160	1.0 (0.9–1.0)	**0.049**
Preoperative secondary lesions	1.1 (0.2–5.3)	0.938		

Significant *p*-values appear in bold. CT: computed tomography, HR: hazard ratio. As only 10 recurrences (events) occurred, it was not possible to include many variables in the analysis to avoid the risk of overfitting. * Analyzed as a continuous variable. If age and lesion size are dichotomized (≤60, >60 and ≤5 cm, >5 cm), the HR are 1.0 (95% CI 0.3–3.6, *p* = 0.995) and 0.1 (95% CI 0.1–1.0, *p* = 0.050), respectively.

## Data Availability

The original contributions presented in this study are included in the article/Supplementary Materials. Further inquiries can be directed to the corresponding author.

## Supplementary Materials

The following supporting information can be downloaded at: https://www.mdpi.com/article/10.3390/pathogens15070756/s1, Table S1: Postoperative outcomes of the patients who presented a recurrence during the follow-up (n = 10); Table S2: Comparison of patients after propensity score matching based on age, preoperative albendazole, lesion size, presence of extrahepatic metastasis, and number of resected segments.
