# Supplementary material for: Long-Term Outcomes After Hepatectomy for Alveolar Echinococcosis in Immunosuppressed Patients
Source: Pathogens. 2026 Jul 19;15(7):756. doi: 10.3390/pathogens15070756 (PMC13414700; doi:10.3390/pathogens15070756)
Supplement: Supplementary file 1 [file pathogens-15-00756-s001.zip › pathogens-4394326-supplementary.pdf]

Supplementary Table 1. Postoperative outcomes of the patients who presented a recurrence during the follow-up (n=10).

	Median or number	IQR or percentage
Largest lesion on pathology, cm	9	5-13
Number of lesions	1	(1-1)
Postoperative albendazole	7	70%
PNM stages		
I	6	60%
II	1	10%
III	1	10%
IV	2	20%
R0/R1	4/6	40%/60%
Complications		
Minor	1	10%
Major	4	40%
90-day mortality	0	-
CCI	8.7	0-33.5
Biliary leak	3	30%
Hemorrhage	1	10%
SSI	0	-
Liver failure	1	10%
Length of stay, days	10	9-13
Reoperation	1	10%
Readmission	2	20%

CCI: comprehensive complication index, SSI: surgical site infection, IQR: interquartile range.
R0: complete resection with microscopically negative margins, R1: complete macroscopic resection with microscopically positive margins.

Supplementary Table 2. Comparison of patients after propensity score matching based on age, preoperative albendazole, lesion size, presence of extrahepatic metastasis, and number of resected segments.

	Immunosuppressed n=46	Non immunosuppressed n=46	P-value
Age, years	62 (50-72)	66 (51-72)	0.611
Body mass index, kg/m^2	25 (21-28)	24 (21-27)	0.647
ASA scores			0.819
I-II	32 (70%)	33 (71%)	
III-IV	14 (30%)	34 (29%)	
Preoperative albendazole	17 (37%)	22 (48%)	0.151
Largest AE lesion on CT, cm	6 (3-9)	6 (4-8)	0.707
Synchronous extrahepatic lesion	8 (17%)	5 (11%)	0.564
Major hepatectomy	23 (50%)	26 (57%)	0.531
Number of resected segments	2 (2-4)	2 (2-4)	0.413
Operation time, min	198 (163-280)	242 (162-332)	0.182
Intraoperative blood loss, ml	450 (388-750)	450 (200-950)	0.455
R0 resection	34 (74%)	33 (71%)	0.815
Postoperative albendazole	43 (93%)	42 (91%)	0.694
PNM stages			0.702
I	20 (43%)	18 (40%)	
II	6 (13%)	8 (17%)	
III	12 (27%)	15 (32%)	
IV	8 (17%)	5 (11%)	
Complications	20 (43%)	23 (50%)	0.531
CCI	0 (0-27.6)	4.4 (0-35.7)	0.439
Length of stay, days	10 (6-14)	11 (8-18)	0.313
Recurrence	4 (9%)	2 (4%)	0.398

ASA: American Society of Anesthesiologists, AE: alveolar echinococcosis, CT: computed tomography, CCI: comprehensive complication index.
